# Chronic Cough as the First Clinical Sign of Fabry Disease: A Case Report

**DOI:** 10.7759/cureus.65716

**Published:** 2024-07-30

**Authors:** Katarzyna Muras-Szwedziak, Kacper Mazurkiewicz, Leon Pawlik, Krzysztof Kaczmarek

**Affiliations:** 1 Department of Clinical Genetics, Central Clinical Hospital of the Medical University of Lodz, Łódź, POL; 2 Department of Electrocardiology, Medical University of Lodz, Łódź, POL

**Keywords:** alpha-galactosidase a, lysosomal storage disorder, genetic renal diseases, chronic cough, fabry's disease, internal medicine

## Abstract

Fabry disease (FD) is a rare lysosomal storage disorder caused by mutations in the GLA gene, which lead to a deficiency of the alpha-galactosidase A enzyme. Pulmonary involvement is one of the possible manifestations of FD, but it is often overlooked and is rarely the only clinical presentation. Chronic cough is an uncommon and nonspecific symptom of pulmonary involvement in FD. Here, we report a case of a 46-year-old non-smoker, Caucasian male who presented to a general practitioner with chronic cough without a significant medical history. The patient was referred to our hospital after routine blood tests revealed elevated creatinine levels. As his cousin had end-stage chronic kidney disease due to FD, we performed a fluorometric assay of the alpha-galactosidase A activity in dried blood spots, which showed abnormal results. Eventually, genetic testing revealed a mutation in the GLA gene. As respiratory symptoms persisted during hospitalization, spirometry was performed, revealing an obstructive pattern. Furthermore, bronchoscopy showed nonspecific bronchial inflammation. Additionally, end-stage renal disease and hypertrophic cardiomyopathy were diagnosed. The patient was put on enzyme replacement therapy, and underwent kidney transplantation. Despite all these procedures, we did not observe any improvement in his cough. This case highlights that chronic cough may be an important clue for pulmonary involvement in FD and should prompt further evaluation in patients with other features suggestive of FD. Early diagnosis and treatment are essential for improving the outcome and quality of life in patients with FD.

## Introduction

Fabry disease (FD) is a rare X-linked lysosomal storage disorder caused by mutations in the GLA gene, which encodes the alpha-galactosidase A enzyme. This enzyme is responsible for the degradation of globotriaosylceramide (Gb3) and other glycosphingolipids in various tissues. The deficiency or dysfunction of alpha-galactosidase A leads to the accumulation of Gb3 and other substrates in lysosomes, resulting in cellular dysfunction and organ damage [1,2]. The incidence of FD in White males ranges from 1:17,000 to 1:117,000 [3].

Clinical manifestations of FD can vary widely. Early symptoms, in order of decreasing prevalence, typically include neuropathic pain, hypohidrosis, gastrointestinal disturbances, angiokeratomas, and corneal opacities. As the disease progresses, patients may develop more serious complications, with cardiomyopathy, left ventricular hypertrophy, and arrhythmias being the most frequently observed, followed by renal involvement and stroke. Males typically experience an earlier onset and a more severe disease course compared to females, who exhibit greater phenotypic variability, ranging from asymptomatic to severely affected [4,5].

The onset and severity of symptoms vary depending on the type and location of the GLA mutation, the residual enzyme activity, and other modifying factors [5,6]. FD can present at any age, from childhood to adulthood, and has a wide phenotypic spectrum [7]. There is a classical form and non-classical (or atypical) form of the disease. Atypical FD is characterized by a residual alpha-galactosidase A activity, resulting in a milder clinical phenotype compared to the classical form. This residual enzyme activity often leads to later disease onset, typically in the fourth to sixth decade of life, and manifestations that may be confined to a single organ system. The most commonly reported atypical manifestation is the cardiac variant, although other organ-specific variants exist [2,4].

Pulmonary involvement in FD, as it is less common than other organ manifestations, can be easily overlooked due to its non-specific symptoms and signs [8]. In contrast to other systems, the exact prevalence of pulmonary involvement is not yet fully established and varies between studies [9,10]. The most common pulmonary finding in FD is obstructive lung disease with possible presence of restrictive lung disease and interstitial lung disease in later stages of the disease [9]. Chronic cough in the absence of any other symptoms of FD is an uncommon clinical phenomenon. It may be related to Gb3 accumulation in airway epithelium, smooth muscle cells, and pulmonary arterioles, causing inflammation and fibrosis [11-14].

A definitive diagnosis of FD relies on demonstrating a low alpha-galactosidase A enzyme activity in white blood cells or blood plasma. When it is not possible to access enzyme testing or genetic analysis, a skin or kidney biopsy can be used to identify the disease. The biopsy would reveal characteristic glycolipid deposits within the tissue. When examining a kidney biopsy under an electron microscope, one can identify distinctive concentric layers of inclusions, also known as zebra bodies [3].

The treatment of FD should involve enzyme replacement therapy (ERT) in conjunction with additional therapies that target the symptoms resulting from tissue damage and aim to prevent further, non-specific tissue injury. Currently, two forms of ERT exist for FD: agalsidase alfa and agalsidase beta. Recommendations regarding when to initiate ERT in pediatric and adult patients with Fabry disease have been published, but it is important to note that treatment should only be initiated after a definitive diagnosis of the disease has been confirmed [6,15].

## Case presentation

We report a case of a 46-year-old Caucasian male who presented with chronic cough of several years as the sole reported clinical symptom. He had no history of smoking, asthma, gastroesophageal reflux disease (GERD), allergies or respiratory infections. He denied any chest pain, shortness of breath, or wheezing. He reported no unintentional weight loss, fever, or night sweats, and his past medical history was uneventful. He worked as a farmer, with no known relevant environmental exposures, and was not taking any medications.

**Figure 1:**
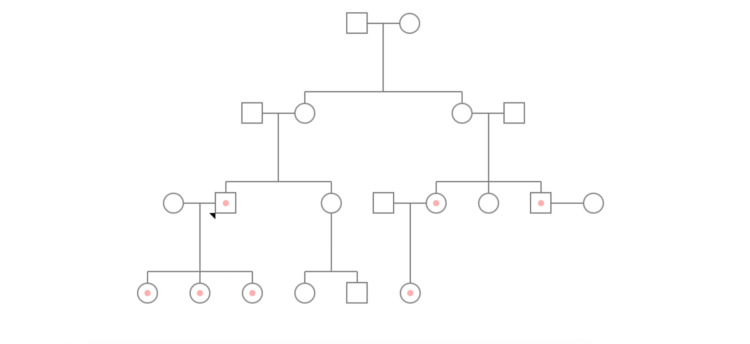
Familial pedigree of the reported case Pink dots represent confirmed c.520T>C (p.Cys174Arg) mutation in GLA gene. The black triangle indicates the patient.

Laboratory tests showed a markedly elevated creatinine level (5.87 mg/dL). Additionally, the patient’s family history was significant for FD; his male cousin on the mother’s side was diagnosed with FD, at age of 17, progressing to end-stage chronic kidney disease (CKD) and ultimately undergoing a kidney transplant (Figure 1). The patient was referred to our hospital for further evaluation.

On admission, the patient’s physical examination was normal, except for a high blood pressure of 176/100 mmHg. Abdominal ultrasonography was performed and revealed small kidneys with thinning of the parenchyma and increased echogenicity. Moreover, the differentiation between the renal cortex and medulla was poor. A cyst measuring 9 mm in diameter was observed in the left kidney, but there were no other focal lesions. No abnormalities were detected in other abdominal organs.

His laboratory test results are shown in Table 1. Other parameters were in the normal ranges.

**Table 1:** Laboratory test results upon admission and one year after the kidney transplant and two years after the start of ERT CK, creatine kinase; ERT, enzyme replacement therapy

Laboratory test	Baseline	One year after the kidney transplant and two years after the start of ERT	Reference range
Blood urea nitrogen	131.06 mg/dl	43.72 mg/dl	0-50 mg/dl
Uric acid	7.3 mg/dl	6.9 mg/dl	3.4-7.0 mg/dl
Creatinine	6.01 mg/dl	1.47 mg/dl	0.7-1.2 mg/dl
CK-MB	8.4 ng/ml	10.1 ng/ml	<4.94 ng/ml
CK-total	191 U/I	78 U/I	<171 U/I
Troponin I	132 ng/l	133 ng/l	<14 ng/l

Due to unknown etiology of CKD and a high suspicion of FD, a fluorometric assay of the alpha-galactosidase A activity in dried blood spots (DBS) was carried out, which showed no activity of the enzyme (0.0 umol/l/h; cut-off value: >2.8 umol/l/h). This test confirmed the diagnosis of FD; furthermore, genetic testing revealed c.520T>C (p.Cys174Arg) mutation in the GLA gene. As the renal failure was consistent with organ involvement in FD, a kidney biopsy was waived.

In a thorough interview focused on FD symptoms, the patient reported recurrent acroparesthesia with burning sensations in his hands that occurred only during febrile infections. The symptom relented with fever and had never occurred without fever. That finding is very characteristic and stays in accordance with some reports in the literature [16]. The patient underwent further investigations to assess the extent of other organs' involvement in FD.

**Figure 2:**
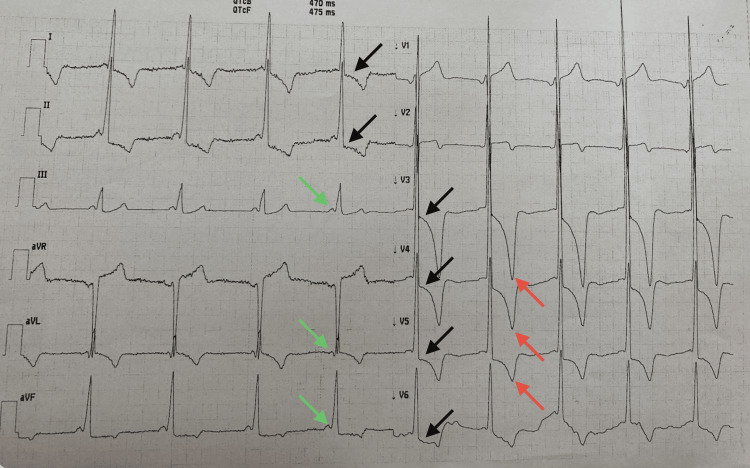
ECG on admission The ECG revealed diffuse ST-segment depressions (black arrows), deep inverted T (red arrows) waves and a shortened PR interval (green arrows), which is a characteristic feature of ECG in FD patients.

Holter ECG revealed ST-segment depressions, deep inverted T waves, a tendency toward bradycardia and a shortened PQ interval, which is a characteristic feature of ECG in FD patients (Figure 2).

Echocardiography showed concentric left-ventricular hypertrophy, which is consistent with FD cardiomyopathy [17]. Brain magnetic resonance imaging revealed ischemic lesions consistent with FD. However, the neurological examination showed no significant abnormalities.

**Figure 3:**
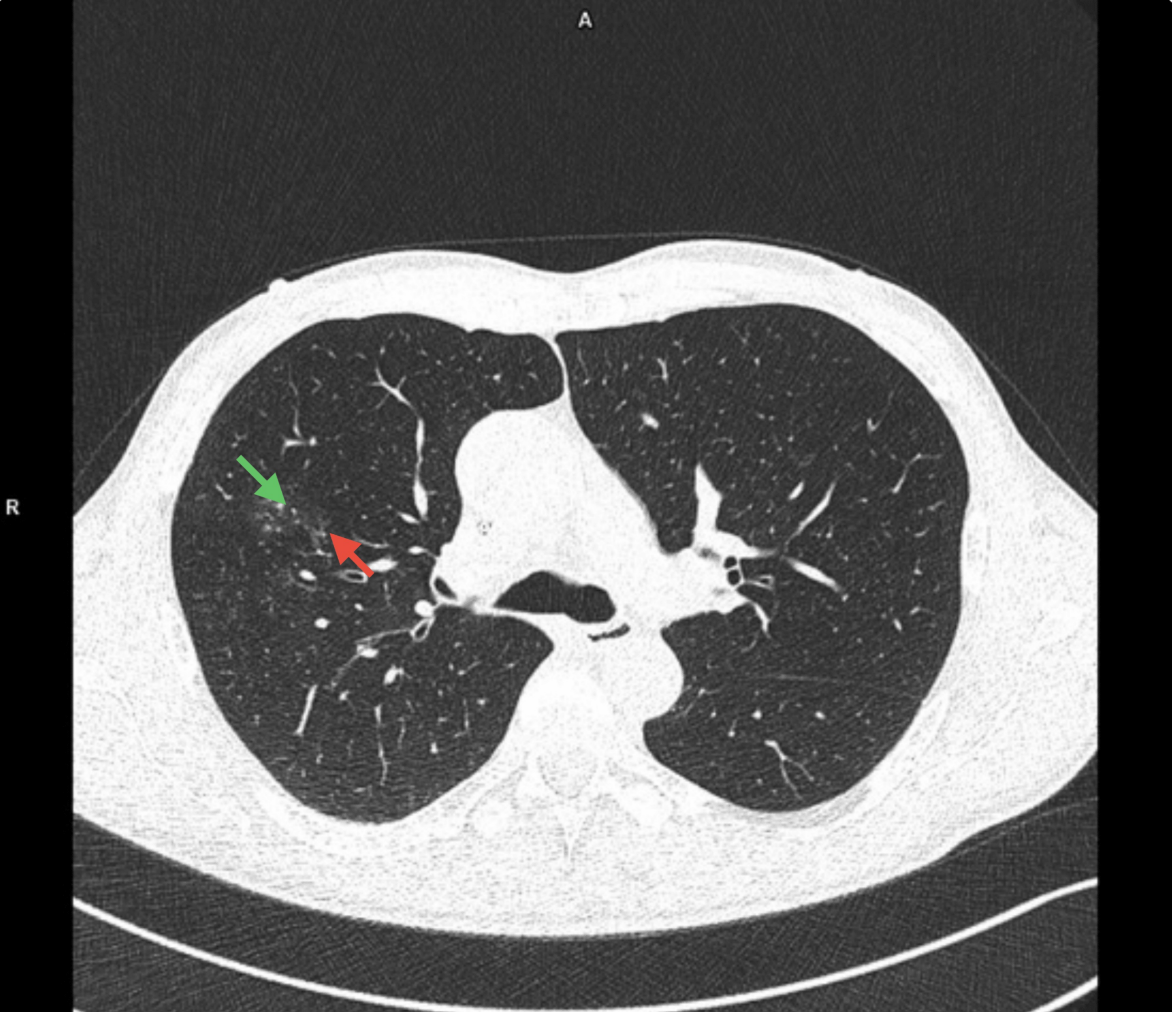
High-resolution CT of lungs High-resolution CT revealed peribronchial ground-glass opacities (green arrow) and tree-in-bud sign (red arrow) in second and third right lung segments.

The pulmonary function was evaluated using spirometry, revealing evidence of irreversible obstruction, characterized by a forced expiratory volume in one second (FEV1) of 2.44 l, a forced vital capacity (FVC) of 4.09 l, and an FEV1/FVC ratio of 60%. His chest X-ray was normal, with no signs of infection, congestion, or fibrosis. High-resolution computed tomography showed peribronchial ground-glass opacities and tree-in-bud sign in second and third right lung segments with a possible inflammatory origin. There were also signs of basal bilateral pulmonary fibrosis (Figure 3).

Diagnostic bronchoscopy was performed and showed signs of chronic bronchial inflammation. A bronchoaspirate sample was collected, and subsequent bacterial cultures, along with *Mycobacterium tuberculosis*-specific PCR analysis, did not reveal any evidence of infection.

Based on the history (no atopy, allergies, smoking, or medication use), physical examination, imaging (chest X-ray and CT scan), laboratory studies (normal inflammatory markers and negative bacterial cultures from bronchoaspirate), and additional tests (normal endoscopy), we were able to rule out the most common causes of chronic cough, including infection, asthma, chronic obstructive pulmonary disease, cancer, medication side effects, GERD and interstitial lung disease. We decided to further examine bronchial tissues (aquired during bronchoscopy) via electron microscopy. This test revealed the presence of zebra-like cytoplasmic inclusions in the bronchial epithelium and pulmonary capillaries endothelium, typical for FD. These examinations were conducted at an external clinic, and despite our efforts, we have been unable to obtain the original results.

The patient started enzyme replacement therapy (ERT) with agalsidase alfa (Replagal) at a dose of 0.2 mg/kg every other week within a month of arriving at our hospital. As a part of a standard procedure for patients that receive ERT in our clinic, the patient had a central venous catheter implanted. Due to the progressive CKD, peritoneal dialysis was introduced. He subsequently underwent a renal transplant in 2022. After one year, the transplanted kidney remained fully functional.

Following the patient's diagnosis and subsequent family screening, his daughter was also found to have FD, and currently receives ERT after initially presenting with acroparesthesia in the feet. The patient's sister also has FD, but details regarding her disease course and its presentation are unavailable.

His chronic cough did not improve, and to this day, he continues to report cough as his most bothersome symptom.

## Discussion

To the best of our knowledge, this is the first case report of a patient with FD who presented with chronic cough as the first and only reported clinical symptom. While cough has been documented in FD, those cases typically presented with other FD-related symptoms or known comorbidities. For instance, Wang et al. described a 47-year-old woman with FD who developed a dry, non-productive cough, but she had a prior diagnosis of Fabry disease and a history of cerebrovascular events [12].

FD is known for its atypical manifestations, affecting a wide range of organs, including the skin, kidneys, heart, lungs, and nervous system. This diversity in organ involvement can lead to misdiagnosis, such as systemic connective tissue diseases or systemic vasculitis [18,19]. However, FD can initially present with symptoms related to just one organ system, which is seen, for example, in the late-onset variant of FD [20].

There is emerging data on pulmonary involvement in lysosomal storage disorders [10]; however, in FD, it is often overlooked or misdiagnosed and is rarely the only clinical presentation [21]. According to one study, FD exhibits bronchial obstruction in 46% of cases, coupled with an annual average decline of 29 ml in FEV1 [2]. Additionally, a separate investigation revealed that the rate of lung function decline was significantly accelerated in male patients, particularly in those undergoing ERT [22].

The pathophysiology of chronic cough in FD is not fully understood, but it may be related to the accumulation of Gb3 in the respiratory epithelium and smooth muscle cells. This may lead to an increased production of pro-inflammatory cytokines, chemokines, and growth factors, which may induce airway inflammation, bronchospasm, fibrosis, and remodeling [23,24]. The presence of zebra-like bodies in the bronchial epithelium of our patient may in fact be a source of chronic cough in this case.

The treatment of FD consists of ERT, which aims to replace the deficient enzyme and reduce the accumulation of Gb3 [6]. There is evidence that the initiation of ERT can stop Gb3 accumulation in tissues [25] and slow down the progression of the disease, especially when treatment is introduced early [26].

The delay in diagnosis in our patient led to the development of CKD, which eventually required a kidney transplant. This could potentially have been avoided if ERT was administered earlier. It is worth mentioning here that FD is not a contraindication to organ transplantation [27]. The effect of ERT on pulmonary involvement and chronic cough in FD remains unclear [21]. Several studies have yielded conflicting results regarding the impact of ERT on pulmonary function [22,28,29]. In our case, the patient did not experience any improvement in his cough after ERT. This could be attributed to the delayed initiation of ERT and the presence of an irreversible lung damage.

## Conclusions

Our case illustrates that chronic cough may be a sign of pulmonary involvement in FD. It may appear as the first clinical manifestation reported by the patient in the absence of other typical FD symptoms. Its presence paired with unexplained renal impairment or a positive family history should prompt further diagnostic workup for FD. Early diagnosis and intervention are crucial in improving the long-term outcomes and quality of life of individuals with FD.
